# BUILDING RESOURCE NETWORKS IN SERVICE OF OLDER PEOPLE THROUGH STATE ARTS AGENCIES' COMMUNITIES OF PRACTICE

**DOI:** 10.1093/geroni/igz038.115

**Published:** 2019-11-08

**Authors:** Beth Bienvenu

**Affiliations:** National Endowment for the Arts, Washington, District of Columbia, United States

## Abstract

National, state, and local arts networks help build the capacity of public and private sector organizations to serve older adults through quality arts engagement opportunities. The National Endowment for the Arts has worked closely with state arts agencies to build networks through a community of practice to facilitate partnerships with artists, arts organizations, aging services, and the healthcare system. With more than 40 states participating, the initiative has resulted in new state partnerships, new state grant initiatives, and new arts learning programs for older adults. Arts service organizations also have a role to play in this work. For example, the American Alliance of Museums is building a network of museums that will develop and implement high-quality, intensive arts learning opportunities for older adults across the United States. This presentation will address how these networks are helping build capacity across the country to improve the health and well-being of older adults

